# Comorbidity of depression and test anxiety among Vietnamese and Chinese undergraduates: cross-cultural insights from latent profile and network analyses

**DOI:** 10.3389/fpsyg.2025.1535224

**Published:** 2025-05-07

**Authors:** Thi Phuong Thao Quach, RenLai Zhou

**Affiliations:** ^1^Department of Psychology, Nanjing University, Nanjing, China; ^2^Department of Radiology, Nanjing Drum Tower Hospital, The Affiliated Hospital of Nanjing University Medical School, Nanjing, China; ^3^State Key Laboratory of Media Convergence Production Technology and Systems, Beijing, China

**Keywords:** test anxiety, depression, China–Vietnam cross-cultural study, multi-group latent profile analysis, network analysis

## Abstract

**Introduction:**

The comorbidity of test anxiety and depression among Chinese students has been documented in numerous studies. However, despite the high prevalence of test anxiety in Vietnam, no studies to date have examined this issue within the Vietnamese context.

**Methods:**

This study tested measurement invariance, assessed test information, and examined symptom interrelations among 884 Vietnamese and Chinese undergraduates. Data were collected using the Test Anxiety Inventory (TAI) and the Beck Depression Inventory-II (BDI-II), administered in either Vietnamese or Chinese. Analyses were conducted using multigroup latent profile analysis (MLPA) and network analysis.

**Results:**

1. Both Chinese and Vietnamese undergraduates experienced comorbidity of test anxiety and depression, with Chinese students in high test anxiety groups showing a greater risk of depressive tendencies. 2. The core symptoms associated with the test anxiety–depression comorbidity differed between the two cultural groups. 3. While the network structures of the high-test-anxiety–high-depression groups showed some similarities across cultures, significant differences were observed in the low-to-moderate test anxiety and moderate depression groups.

**Discussion:**

These findings underscore cultural nuances in the recognition and expression of test anxiety and depression among Vietnamese and Chinese undergraduates. The results highlight the importance of culturally sensitive approaches in cross-cultural mental health research and intervention design.

## 1 Introduction

Diagnostic comorbidity of anxiety and depression refers to the co-occurrence of symptoms from both conditions, where neither is clearly predominant or severe enough to justify a diagnosis on its own (World-Health-Organization, 2016). Research shows that depressive and anxiety symptoms are highly prevalent across countries worldwide (American-Psychiatric-Association, 2022; Beuke et al., 2003; Yan et al., 2023), and their comorbidity is common across all categories of psychiatric diagnoses (Brown et al., 2001), with detection rates reaching 45.7%–70.0% (Fava et al., 2000; Kircanski et al., 2017). Anxiety-depression comorbidity is characterized by overlapping symptoms of both conditions (Means-Christensen et al., 2006), including, but not limited to, restlessness, which refers to a state of agitation or an inability to relax; difficulty concentrating, which involves trouble focusing on tasks or thinking clearly; sleep disturbances, such as problems with falling asleep, staying asleep, or sleeping excessively; fatigue or low energy, marked by persistent tiredness or a lack of motivation despite rest; irritability, which is an easily triggered frustration or annoyance, often without significant causes; worry, which is an excessive concern or fear about potential negative outcomes; tearfulness, involving frequent crying or feeling emotionally overwhelmed; and low self-esteem, which involves a poor self-image or feelings of inadequacy (American-Psychiatric-Association, 2022; World-Health-Organization, 2016). The occurrence of anxiety–depression comorbidity not only harms patients’ physical and mental health but is also associated with higher relapse rates of depression, suicide rates, and disability, along with longer illness durations and relatively poor intervention outcomes (American-Psychiatric-Association, 2022; Kircanski et al., 2017; Yan et al., 2023). Therefore, a better understanding of the interaction between anxiety and depressive symptoms can help reduce the burden caused by their comorbidity (Colovic et al., 2016; Mihić et al., 2023).

In traditional research, the comorbidity of anxiety and depression is typically examined within the context of generalized anxiety disorder (GAD) and major depression. This approach allows for a broader scope in expanding the sample size. Some studies even include participants from various age groups and those confirmed to have anxiety or depression in their research samples (Beuke et al., 2003; Fava et al., 2000). However, Fava et al. (2000) and Kircanski et al. (2017) explicitly noted that individuals with comorbid anxiety and depression tend to experience their first episode at a younger age than those with only anxiety or only depression. This underscores the need for early screening for anxiety–depression comorbidity (Fan et al., 2019; Lowe, 2017).

Test anxiety refers to the physiological and behavioral responses exhibited by individuals in evaluative situations, accompanied by concerns about potential negative outcomes or failure (Hou et al., 2025). As a form of trait anxiety triggered by testing situations, it is one of the most common psychological distress experiences faced by students in Vietnam, China, and worldwide (Fei et al., 2018; Qiong and Ren-lai, 2019; Thảo and Ái, 2021). Given its shared characteristics with trait anxiety, test anxiety may also be associated with an increased risk of comorbidity with depression (Akinsola and Nwajei, 2013). In China, individuals with high test anxiety have been found to exhibit specific processing biases toward negative information (Hou et al., 2025; Shi et al., 2017) and demonstrate general deficits in inhibitory control (Zhang et al., 2019) which is similar to the characteristics observed in individuals with depression (Beuke et al., 2003).

To our knowledge, research on the comorbidity of test anxiety and depression remains limited, with no studies involving cross-cultural samples. In contrast, cross-cultural research on general anxiety–depression comorbidity is more developed (Mihić et al., 2023; Van den Bergh et al., 2020; Schlechter et al., 2020). For example, Van den Bergh et al. (2020) studied 11,647 participants worldwide via network analysis to explore the relationships among stress, anxiety, and depression. They ultimately identified panic, worry, worthlessness, hopelessness, and meaninglessness as key mediating symptoms. Mihić et al. (2023) conducted a network analysis with 874 participants from three neighboring countries—Italy, Croatia, and Serbia. Their findings revealed similarities in the structure and characteristics of anxiety and depression across these countries, although differences emerged in the connections between symptoms and the strength of those connections. In contrast, Schlechter et al. (2020) reported significant differences in the symptom networks and characteristics of anxiety and depression between native Germans and refugees living in Germany, particularly regarding the core symptoms of losing interest and feeling depressed. These differences may arise from cultural diversity or be influenced by the unique living conditions of refugees. Furthermore, it is important to emphasize that in many cultures where suffering is commonly viewed as an unavoidable part of life, individuals often express these problems indirectly through verbal cues, which can lead to clinical presentations that are predominantly characterized by physical symptoms. Cross-cultural research, therefore, not only requires ensuring equivalence in research instruments but also necessitates the use of various research methods to explore symptom characteristics in as much detail as possible (Van den Bergh et al., 2020; Schlechter et al., 2020).

When researchers investigate the mechanisms of anxiety–depression comorbidity, they often hypothesize that these two disorders share a common cause, such as neuroticism or environmental stressors (Van den Bergh et al., 2020), suggesting that this comorbidity may develop from the exacerbation of these factors. These studies primarily use the classical test theory (CTT) approach with self-report measures (Akinsola and Nwajei, 2013; Kircanski et al., 2017; Mihić et al., 2023; Van den Bergh et al., 2020; Schlechter et al., 2020). The classical test theory (CTT) adopts a variable-centered perspective, assumes homogeneity among participants within a group, and treats all measured items as equally important, thereby providing generalized information efficiently. However, a major limitation of this approach is its tendency to overlook individual heterogeneity (Schlechter et al., 2020). In contrast, latent profile analysis (LPA), which is also based on the common cause hypothesis, offers an individual-centered research method that addresses within-group heterogeneity by clustering individuals with similar responses into the same group, thereby avoiding oversimplification. Multi-group latent profile analysis (MLPA) allows for direct comparisons of latent variable response patterns across multiple groups, ensuring equivalence in measurement instruments and effectively handling cross-cultural data (Fan et al., 2019). The network theory of psychopathology offers a different perspective on the relationship between anxiety and depression, suggesting that mental disorders are composed of networks of symptoms that reinforce and causally relate to each other (Van den Bergh et al., 2020; Schlechter et al., 2020). Within this network framework, comorbidity may arise from network communities where several symptoms (“nodes”) of anxiety and depression mutually influence and interact with each other (“edges”), forming strongly interconnected network communities (Van den Bergh et al., 2020; Schlechter et al., 2020). In a cross-cultural research context, network analysis offers a more comprehensive understanding of the differences and commonalities in mental health comorbidity across cultures.

Despite geographical proximity, many countries exhibit cultural differences in the prevalence, manifestation, and symptom characteristics of depression and test anxiety (Lowe, 2017; Mihić et al., 2023). These differences may stem from variations in measurement tools (Mihić et al., 2023), socioeconomic factors (King et al., 2023), national education policies (Bodas and Ollendick, 2005), and genuine differences influenced by cultural values and societal behavioral norms (Mihić et al., 2023). In the context of research on the comorbidity of test anxiety and depression in China and Vietnam, earlier studies reported a positive correlation between test anxiety and depression among student populations in both countries (Nguyen et al., 2013; Thành et al., 2021). While the comorbidity of test anxiety and depression has been confirmed among Chinese students (Shi et al., 2017; Wei et al., 2016), research on test anxiety in Vietnam is relatively new, and no studies have yet examined the comorbidity of test anxiety and depression in this context. Notably, the prevalence of test anxiety among Vietnamese students is relatively high—exceeding 50%—and significantly higher than that reported among Chinese students (approximately 22%–30%) (Hoa, 2021; Qiong and Ren-lai, 2019; Thảo and Ái, 2021). These findings suggest that Vietnamese students may be at risk of testing for anxiety–depression comorbidities. Furthermore, within the cultural contexts of China and Vietnam, the manifestations of test anxiety and depression may differ, and the nature of their comorbidity could vary accordingly.

This study aims to compare the comorbid symptoms of test anxiety and depression among Chinese and Vietnamese undergraduates. Using the Test Anxiety Inventory (TAI) and the Beck Depression Inventory-II (BDI-II), we apply multi-group latent profile analysis (MLPA) and multi-group network analysis to address the following research questions: (1) How do test anxiety and depression manifest conceptually within the cultural contexts of China and Vietnam, and what are the differences in the latent comorbid groups between the two countries?; (2) What is the overall prevalence and status of test anxiety and depression in both China and Vietnam?; and (3) How do test anxiety and depressive symptoms interact in each country, and what are the core comorbid symptoms? What are the degrees of influence and patterns of symptom transmission between them?

## 2 Methods

### 2.1 Sample

The study collected data from 1,081 students at Nanjing University (February–July 2019) and 956 students across eight universities in northern Vietnam (April 2018–March 2019). Responses from nonundergraduates, incomplete data, or participants outside the 17–24 age range were excluded. A total of 442 valid Chinese and 844 valid Vietnamese responses with complete TAI and BDI-II data were retained. To address sample size differences, 422 Vietnamese participants were randomly selected (see Table 1).

**Table 1:** Characteristics of the Vietnamese and Chinese undergraduate samples.

Group	Age (M ± SD)	Gender		Academic year				Total
		Female	Male	1	2	3	4	
Vietnamese	20.29 ± 1.1	313	129	57	181	131	73	442
Chinese	19.99 ± 1.4	313	129	157	118	91	76	442

The Shapiro–Wilk test and t tests revealed no significant differences between the selected subsample and the original Vietnamese dataset in terms of age, gender, TAI, or BDI-II scores (*F* < 0.576; *p* > 0.464), confirming the sample’s representativeness.

### 2.2 Measures

#### 2.2.1 Test anxiety

The Test Anxiety Inventory (TAI) measures two key dimensions of test anxiety: worry (TAIW), which refers to cognitive concerns about test outcomes, and emotionality (TAIE), which refers to autonomic arousal (Spielberger and Vagg, 1995). It is an internationally validated instrument (Sharma and Sud, 1990), consisting of 20 items on a 4-point Likert scale, with item 1 reverse-scored. Total scores range from 20 to 80, with higher scores indicating greater test anxiety. TAI is suitable for secondary school students and above, and in the Vietnamese and Chinese cultural context, it has been shown to have strong reliability and validity (Cronbach’s *α* > 0.80) (Thảo et al., 2019; Wang, 2003). In this study, the internal consistency was excellent, with Cronbach’s α = 0.94 for the Chinese version and α = 0.92 for the Vietnamese version.

#### 2.2.2 Depression

The Vietnamese and Chinese culturally adapted versions of the Beck Depression Inventory-II (BDI-II) were used (Khanh, 2017; Zhang et al., 1990). The BDI-II is an internationally validated instrument and can distinguish clearly between depression and anxiety (Riezen and Seigal, 1988). The BDI-II consists of 21 items rated on a 4-point Likert scale, with total scores ranging from 0 to 63, where higher scores indicate greater levels of depression. In both Vietnamese and Chinese contexts, the BDI-II has demonstrated strong reliability and validity, with Cronbach’s α > 0.89 (Khanh, 2017; Zhang et al., 1990). In this study, the internal consistency was excellent, with Cronbach’s α = 0.89 for the Chinese version and α = 0.91 for the Vietnamese version.

### 2.3 Multi-group latent profile analysis

First, single LPAs with 1 to 5 solutions were conducted separately for the Vietnamese and Chinese samples to determine the number of test anxiety–depression comorbidity profiles in each group. The number of profiles was based on statistical criteria, practicality, and interpretability of the extracted profiles. Fit indices were compared to ensure that the optimal number of profiles was consistent across both countries. The key indicators for evaluating LPA model fit include the Akaike information criterion (AIC), Bayesian information criterion (BIC), entropy, and bootstrapped likelihood ratio test (BLRT), with lower AIC and BIC values, higher entropy, and a significant BLRT indicating better model fit (Fan et al., 2019; Olivera-Aguilar and Rikoon, 2017).

In the second step, the response patterns of students from the Vietnamese and Chinese samples on test anxiety–depression comorbidity were examined to determine whether they were consistent across the two samples. This step involved conducting and comparing a series of multi-group LPAs, including unrestricted, semiconstrained, and fully constrained MLPA models, by combining all samples into a single data run with “country” as the grouping variable. In the unrestricted model, the within-profile means and variances of anxiety–depression comorbidity, along with the profile sizes, varied across the two samples, indicating that the measurement invariance assumption did not hold. In the semiconstrained model, the profile sizes were allowed to differ, but the within-profile means and variances of anxiety–depression comorbidity were constrained to be equal across samples. For the fully constrained model, both the profile sizes and the within-profile means and variances were set to be equivalent, satisfying the measurement invariance assumption (Fan et al., 2019).

Previous research suggests that if the semiconstrained model provides the best fit, the conceptualisation of the two cultures is equivalent and supports the validity of cross-cultural comparisons in the study results (Fan et al., 2019; Olivera-Aguilar and Rikoon, 2017).

### 2.4 Network analysis

We used Spearman correlations to assess the relationships (edges) between pairs of symptoms (nodes) in the full sample and subgroups (based on the latent profile analysis). The Fruchterman-Reingold (FR) algorithm and spring layout were applied to generate symptom networks. In the FR algorithm, the node (symptom) with the highest centrality is positioned at the center of the network, and nodes with similar characteristics are placed closer together (Van den Bergh et al., 2020; Schlechter et al., 2020). The bootnet function and the Ising model were used to independently assess network invariance between the test anxiety–depression networks in China and Vietnam by evaluating the strength of connections. Given that closeness and betweenness centralities are less reliable with small sample sizes (Schlechter et al., 2020), this study focused on strength as the primary centrality measure among the three indices. Previous research has suggested that a stability coefficient (CS) for strength centrality between 0.2 and 0.5 indicates moderate network stability, whereas values between 0.5 and 0.7 indicate good stability (Mihić et al., 2023).

All analyses in this study were conducted via SPSS 20, Mplus 7.0, and R 4.0.2 with the bootnet, qgraph, NetworkComparisonTest, and dplyr packages.

## 3 Results

### 3.1 Correlation

The correlation between TAI and BDI-II scores among Vietnamese undergraduates was not significant (*p* = 0.179). In contrast, a significant positive correlation was found between TAI and BDI-II scores among Chinese undergraduates (r = 0.54, *p* < 0.001).

### 3.2 Multi-group latent profile analysis

**Figure 1:**
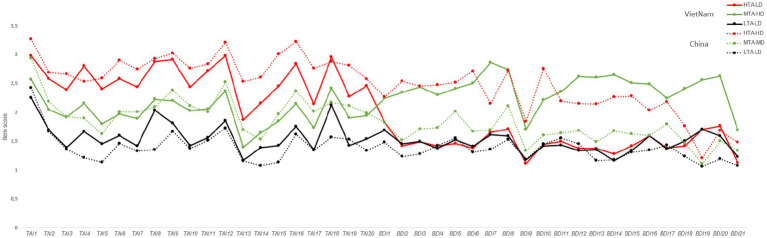
Three different profiles of test anxiety (TAI) and depression (BDI-II) in Vietnamese and Chinese undergraduate samples.

The statistical tests for the independent model fit in China and Vietnam indicated that the AIC and BIC values decreased monotonically as the number of latent profiles increased. In the Vietnamese sample, the entropy value was highest for the three-profile model, suggesting that the classification accuracy was optimal with three profiles. Although the entropy value for the Chinese sample was not highest in the three-profile model, the decreases in the AIC and BIC from three to four or five profiles were much smaller than the decreases observed from one to three profiles. Additionally, the BLRT values for both countries reached significant levels, indicating that the three-profile solution was the most reasonable fit for both Chinese and Vietnamese undergraduates (see Figure 1; Table 2).

**Table 2:** Fit indices for single LPA models across Vietnamese and Chinese samples.

Nation classes	Vietnam				China			
	AIC	BIC	Entropy	BLRT(*p*)	AIC	BIC	Entropy	BLRT(p)
1	43969.742	44305.229			40488.067	40823.557		
2	41719.158	42226.481	0.925	<0.01	36472.433	36979.755	0.967	<0.01
3	40024.034	40703.191	0.962	<0.01	35411.675	36090.832	0.945	<0.01
4	39533.220	40384.212	0.954	<0.01	34782.883	35633.876	0.938	<0.01
5	39145.324	40168.152	0.941	<0.01	34317.235	35340.062	0.946	<0.01

AIC, Akaike information criterion; BIC, Bayesian information criterion; BLRT, bootstrap likelihood ratio test, *p* < 0.05 suggest significantly better performance.

The semiconstrained model for the TAI and BDI-II questionnaires presented the lowest AIC, BIC, and SABIC values (see Table 2). This suggests that the TAI and BDI-II share similar latent structural characteristics within the Chinese and Vietnamese undergraduate samples, indicating that the conceptualisation of the two cultures is equivalent and supporting the validity of cross-cultural comparisons in the study results (Fan et al., 2019; Olivera-Aguilar and Rikoon, 2017) (Table 3).

**Table 3:** Fit indices for multi-group LPA models.

Model	TAI			BDI-II		
	AIC	BIC	SABIC	AIC	BIC	SABIC
Unconstrained	37593.860	38278.038	37823.899	38377.512	39095.181	38618.811
Semiconstrained	36980.433	37592.843	37186.342	34527.056	35168.173	34742.617
Fully constrained	37898.868	38501.709	38101.559	39171.107	39802.655	39383.450

AIC, Akaike information criterion; BIC, Bayesian information criterion; SABIC, Sample-Size Adjusted BIC.

On the basis of the classification results of the latent profile analysis, three latent profiles were plotted, illustrating participants’ scores on the 20 TAI items and 21 BDI-II items (see Figure 1). After performing latent profile analysis (LPA) for both countries, a chi-square test with Fisher’s exact test was conducted to examine whether there were significant differences in the distribution of latent profile classifications between the two countries. The results revealed significant differences in classification between Chinese and Vietnamese participants (Pearson’s chi-square = 450.747, df = 4, *p* < 0.001; Fisher’s exact test *p* < 0.001). Differences in profile size were detected in the Vietnamese sample for high test anxiety–low depression (HTA-LD) 31.9%, moderate test anxiety–high depression (MTA-HD) 16.96%, and low test anxiety–low depression (LTA-LD) 51.13%, and in the Chinese sample for high test anxiety–high depression (HTA-HD) 13.57%, moderate test anxiety–moderate depression (MTA-MD) 39.37%, and low test anxiety–low depression (LTA-LD) 47.05%.

A 2 (nationality: China/Vietnam) × 2 (gender: male/female) × 3 (group: high/moderate/low test anxiety) factorial ANOVA was conducted on the total TAI score, TAI emotionality dimension score (TAIE), and TAI worry dimension score (TAIW). The results revealed that the main effect of gender on TAI, TAIE, and TAIW was not significant [1.043 < *F*_(1, 872)_ < 1.665, 0.197 < *p* < 0.307]. The main effect of nationality on the total TAI score was significant [*F*_(1, 872)_ = 6.427, *p* = 0.011, ηp^2^ = 0.007], indicating that Chinese students had higher overall anxiety levels than Vietnamese students did. However, there were no significant nationality effects on the TAIE or TAIW scores [0.168 < *F*_(1, 872)_ < 2.181, 0.14 < *p* < 0.682].

The group main effects for TAI, TAIE, and TAIW were all significant [552.311 < *F*_(2, 872)_ < 765.158, *p* < 0.001]. The interaction effects between gender and nationality were not significant for TAI and TAIE [0.62 < *F*_(1, 872)_ < 0.857, 0.355 < *p* < 0.431]. However, the interaction effect for TAIW was significant [*F*_(1, 872)_ = 4, *p* = 0.046, ηp^2^ = 0.005]. A simple effect analysis revealed that male students in China reported significantly higher worry levels than their female peers did [*F*_(2, 872)_ = 4.337, *p* = 0.038, ηp^2^ = 0.005]. This sex difference was not observed in the Vietnamese sample. The interaction effect between gender and group was not significant for TAIW [*F*_(2, 872)_ = 1.781, *p* = 0.169, ηp^2^ = 0.004]. However, significant interaction effects were found for TAI and TAIE [3.968 < *F*_(2, 872)_ < 4.963, 0.019 < *p* < 0.026]. A simple effect analysis indicated that in the high test anxiety group, female participants had higher overall anxiety levels (M-TAI = 54.578) than male participants did (M-TAI = 52.256) [*F*_(2, 872)_ = 4.983, *p* = 0.026, ηp^2^ = 0.006]. In the low test anxiety group, female participants had higher emotionality scores (M-TAIE = 13.086) than male participants did (M-TAIE = 12.326) [*F*_(2, 872)_ = 6.313, *p* = 0.012, ηp^2^ = 0.007]. The interaction effects between nationality and group were significant for TAI, TAIE, and TAIW [20.375 < *F*_(2, 872)_ < 29.304, *p* < 0.001]. A simple effect analysis revealed that in the low test anxiety group, Vietnamese students had significantly higher overall anxiety, emotionality, and worry scores than Chinese students did [27.165 < *F*_(2, 872)_ < 43.816, *p* < 0.001]. In contrast, in the high test anxiety group, Chinese students had significantly higher scores for overall anxiety, emotion, and worry than Vietnamese students did [8.403 < *F*_(2, 872)_ < 29.039, *p* < 0.004]. The three-way interaction effects among gender, nationality, and group were not significant for TAI, TAIE, or TAIW [0.009 < *F*_(2, 872)_ < 1.1, 0.333 < *p* < 0.991] (see Table 4).

**Table 4:** Summary of TAI and BDI-II scores (M ± SD) by latent profile groups among Chinese and Vietnamese university students.

Gender nation		Group	TAI			BDI
				Emotionality	Worry	
Vietnam	Male	HTA-LD	49.2 ± 5.6	21.34 ± 2.9	18. 57 ± 3. 06	9.86 ± 5.89
		MTA-HD	41 ± 7.76	17.64 ± 3.3	15. 36 ± 3. 76	30.32 ± 8.8
		LTA-LD	31.32 ± 5.6	13.37 ± 2.7	11. 76 ± 2. 85	8.83 ± 6.42
	Female	HTA-LD	52.02 ± 7.7	21.92 ± 3.48	19. 7 ± 3. 98	9.69 ± 6.19
		MTA-HD	39.89 ± 8.09	16.64 ± 3.86	14. 9 ± 3. 62	29.11 ± 7.01
		LTA-LD	32.75 ± 5.6	14.04 ± 3.07	11.8 ± 2.46	9.6 ± 6.17
China	Male	HTA-HD	55.3 ± 5.5	22.56 ± 2.7	21.94 ± 2.77	24.38 ± 7.356
		MTA-MD	42.75 ± 5.54	17.52 ± 2.79	15.94 ± 2.47	11.83 ± 7.421
		LTA-LD	28.48 ± 4.4	11. 28 ± 2. 03	10. 3 ± 1. 85	7.6 ± 6.487
	Female	HTA-HD	57.14 ± 7.3	23. 68 ± 3. 4	21. 23 ± 3. 81	13.7 ± 7.037
		MTA-MD	41.02 ± 5.2	17. 17 ± 2. 6	14. 6 ± 2. 47	24.98 ± 7.366
		LTA-LD	29.15 ± 4.5	12. 13 ± 2. 4	10. 2 ± 1. 85	6.31 ± 5.458

A 2 (nationality: China and Vietnam) × 2 (gender: male and female) × 3 (group: high/moderate/low test anxiety) factorial ANOVA was conducted on the total BDI-II scores. The results revealed that the main effect of gender was not significant [*F*_(1, 872)_ = 0.029, *p* = 0.865, ηp^2^ < 0.001]; the main effect of nationality was significant, with Vietnamese students (M = 16.235) reporting significantly greater depressive tendencies than Chinese students did (M = 14.799) [*F*_(1, 872)_ = 6.753, *p* = 0.01, ηp^2^ = 0.008]; and the main effect of group was significant [*F*_(2, 872)_ = 271.086, *p* < 0.001, ηp^2^ = 0.383]. Neither the gender–nationality interaction nor the gender–group interaction was significant [0.149 < *F*_(1, 872)_ < 0.29, 0.591 < *p* < 0.861, ηp^2^ < 0.001]; the nationality–group interaction was significant [*F*_(2, 872)_ = 221.536, *p* < 0.001, ηp^2^ = 0.337]. Simple effect analysis revealed that in the Vietnamese sample, the moderate test anxiety group reported significantly greater depressive tendencies than both the high and low test anxiety groups did [*F*_(2, 872)_ = 250.719, *p* < 0.001, ηp^2^ = 0.365], whereas in the Chinese sample, the high test anxiety group reported significantly greater depressive tendencies than did the moderate test anxiety group did, and the moderate test anxiety group reported significantly greater depressive tendencies than did the low test anxiety group did [*F*_(2, 872)_ = 142.155, *p* < 0.001, ηp^2^ = 0.246]. The nationality, gender, and group interaction was not significant [*F*_(2, 872)_ = 2.393, *p* = 0.092, ηp^2^ = 0.005].

### 3.3 Network analysis

**Figure 2:**
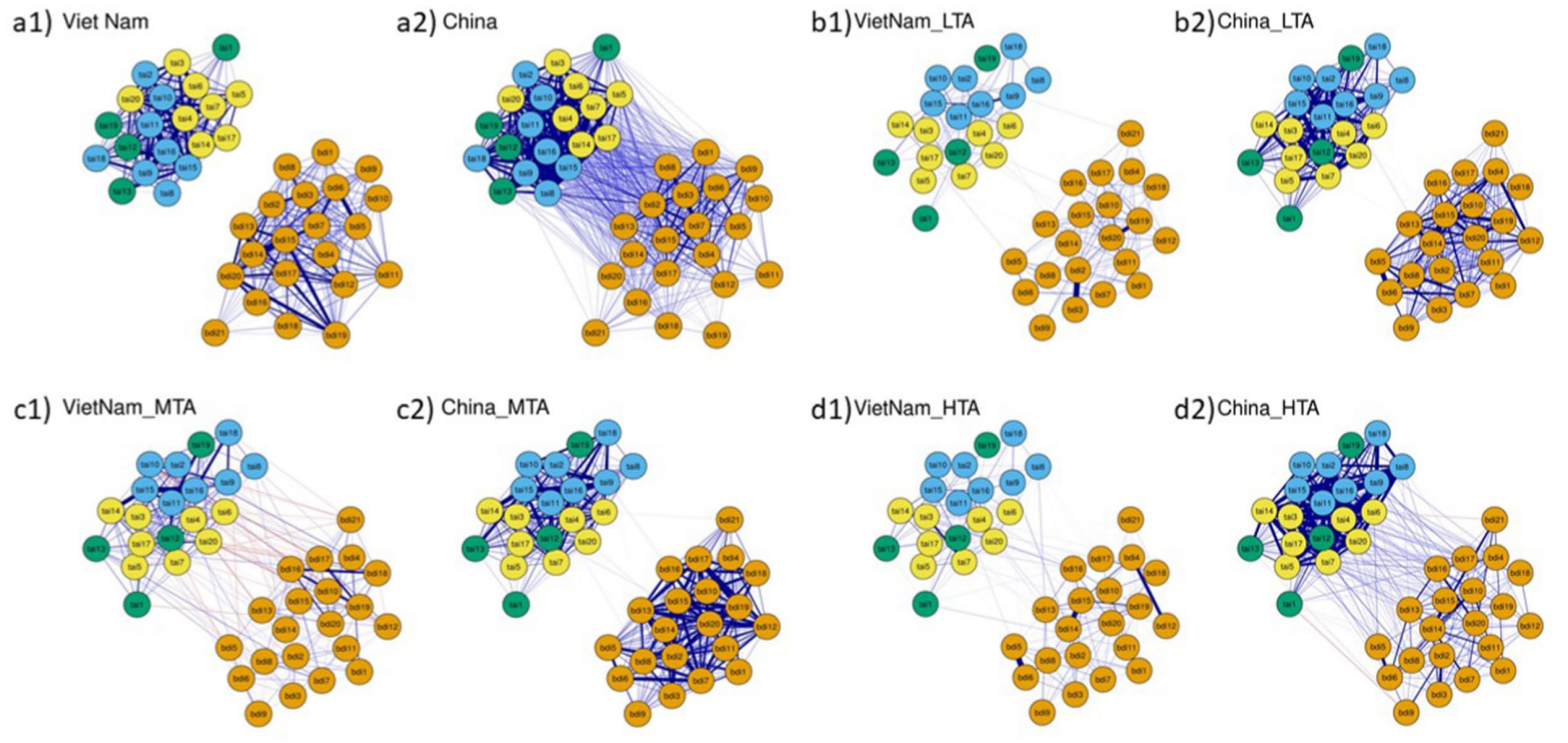
Symptom network of test anxiety and depression based on latent profile categories among Chinese and Vietnamese undergraduates: Full sample and three different profiles of test anxiety (TAI) and depression (BDI-II): **(a1)** Vietnamese Full Sample, **(a2)** Chinese Full Sample, **(b1)** Vietnamese Low Test Anxiety (VietnaM-LTA), **(b2)** Chinese Low Test Anxiety (China_LTA), **(c1)** Vietnamese Middle Test Anxiety (VietnaM-MTA), **(c2)** Chinese Middle Test Anxiety (China_MTA), **(d1)** Vietnamese High Test Anxiety (VietnaM-HTA), **(d2)** Chinese High Test Anxiety (China_HTA). Orange nodes represent depression (BDI-II); yellow nodes represent the test anxiety worry component; blue nodes represent the test anxiety emotionality component; and green nodes represent other test anxiety items. In the Fruchterman-Reingold algorithm, the node with the strongest centrality is placed at the center of the network. The thickness of the edges corresponds to the strength of the association.

Figure 2 presents the visualization of the network models for the overall samples from China and Vietnam, as well as for each country’s subgroups. Each network contains 41 nodes, representing the 20 items from the Test Anxiety Inventory (TAI) and the 21 items from the Beck Depression Inventory-II (BDI-II). In the figure, blue lines indicate positive correlations, and red lines indicate negative correlations; the thicker the line is, the stronger the partial correlation.

The stability of the expected influence strength in the networks for the overall samples and each anxiety–depression group in both countries is acceptable (Vietnam: 0.595, 0.206, 0.28, 0.518; China: 0.751, 0.367, 0.362, 0.438). This suggests that the strength of connections between anxiety and depression symptoms shows some degree of stability and consistency across different cultural contexts and levels of test anxiety. The mean edge weights in the Chinese anxiety-depression networks are notably greater than those in the Vietnamese networks are, indicating stronger connections within the Chinese networks (nonzero edges and mean edge weights in Vietnam: 820 and 0.18, 820 and 0.08, 820 and 0.099, 820 and 0.08; in China: 820 and 0.295, 820 and 0.18, 820 and 0.175, 818 and 0.187). Additionally, the nonzero edge results for both countries indicate that all networks in this study are highly dense.

**Figure 3:**
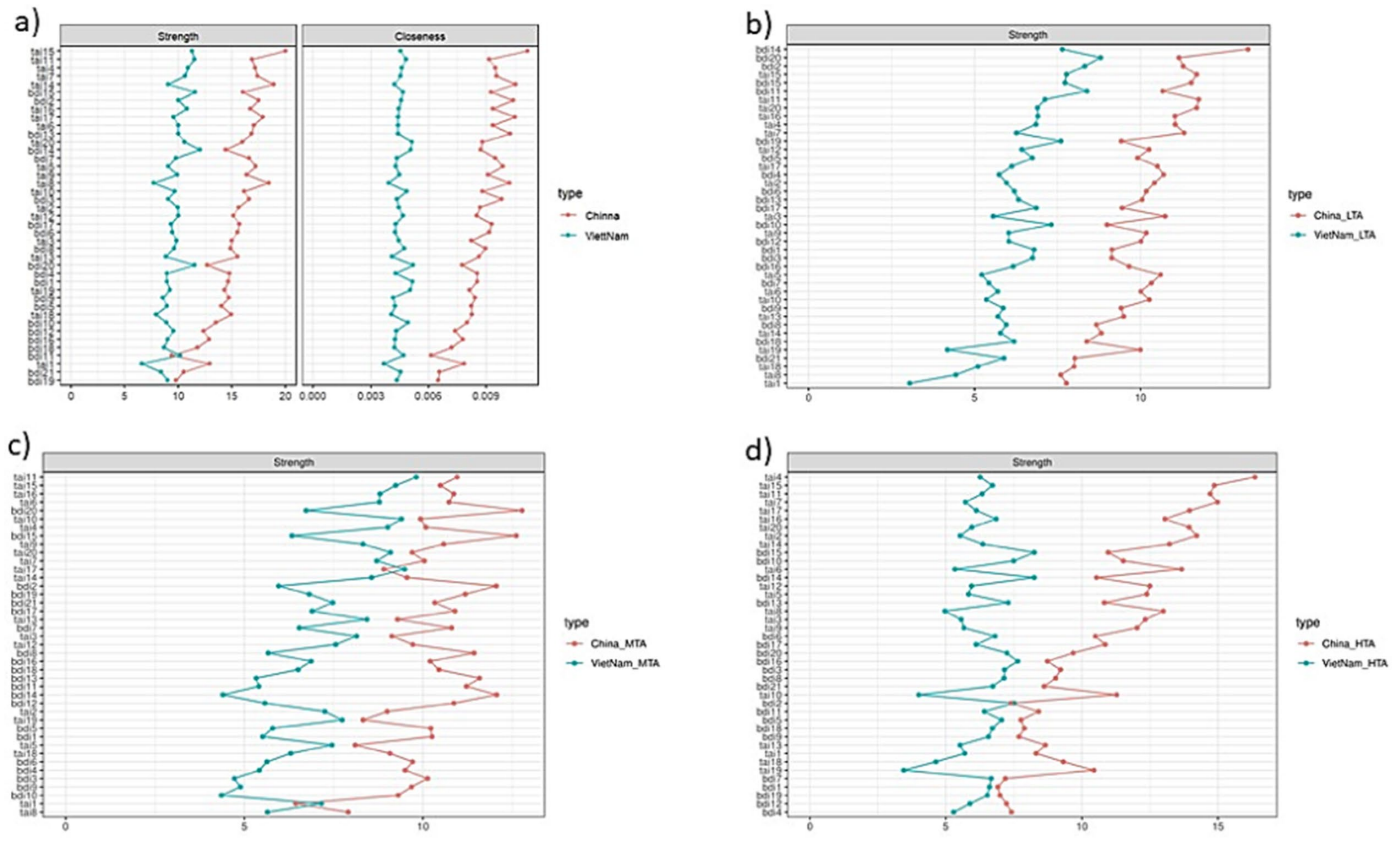
Network centrality indicators of test anxiety and depression based on latent profile categories among Chinese and Vietnamese undergraduates: full sample and three different profiles of test anxiety (TAI) and depression (BDI-II): **(a)** full sample, **(b)** low test anxiety (LTA), **(c)** middle test anxiety (MTA), and **(d)** high test anxiety (HTA).

From the overall perspective of network node centrality metrics, on the one hand, the node centrality strength in almost all of China’s networks is significantly greater than that of Vietnam (see Figure 3). On the other hand, regarding the strength of symptoms (nodes) within the networks, the node centrality metrics exhibit opposite trends in the test anxiety–depression network structures of China and Vietnam (see Figure 3). Specifically, in the overall Vietnamese test anxiety–depression network, BDI-II 14 (feelings of worthlessness) is the most central symptom, followed by BDI-II 15 (feelings of helplessness) and BDI-II 20 (fatigue). TAI 11 (feeling very nervous during exams) is also among the key symptoms. However, these symptoms have weaker centrality in the Chinese network. In contrast, the most central symptom in the Chinese test anxiety–depression network is TAI 15 (feeling panicked during important exams), followed by TAI 14 (feeling defeated during important exams), TAI 8 (nervousness), and TAI 17 (constant thoughts of failure). Further observations revealed that in the low test anxiety–depression group networks, BDI-II 20, BDI-II 11, and BDI-II 2 are key symptoms for Vietnam, whereas BDI-II 14, TAI 11, TAI 20, and TAI 15 are central symptoms for China. In the moderate test anxiety–high depression group networks, Vietnam’s key symptoms are TAI 11, TAI 17, and TAI 10, whereas China’s key symptoms are BDI-II 20, BDI-II 15, BDI-II 14, and BDI-II 2. In the high test anxiety-depression group networks, Vietnam’s central symptoms are BDI-II 15 and BDI-II 14, whereas China’s key symptoms are TAI 4, TAI 7, TAI 15, and TAI 11 (see Figure 3).

Although the connections between nodes in the test anxiety–depression networks of China and Vietnam are not the strongest (see Figure 2), this study focuses on the comorbidity of test anxiety and depression among university students in these two countries. Therefore, the connecting edges constitute the primary subject of analysis in this paper. Overall, the connections between test anxiety and depressive symptoms are less dense among Vietnamese university students than among their Chinese counterparts. However, there are notable differences in the strength and patterns of associations at various levels of test anxiety and depression between the two student groups (see Figure 2). Specifically, in the low test anxiety-depression group, the edge strengths for Vietnamese students are as follows: TAI 14 – BDI 5 (r = −0.2), TAI 2 – BDI 21 (r = −0.19), TAI 20 – BDI 10 (r = −0.19), and TAI 20 – BDI 1 (r = 0.18). In comparison, the edge strengths for Chinese students in the low test anxiety-depression group are as follows: TAI 16 – BDI 2 (r = 0.2), TAI 10 – BDI 1 (r = 0.16), TAI 17 – BDI 13 (r = 0.16), and TAI 19 – BDI 14 (r = 0.16). For the moderate test anxiety-depression group, the edge strengths for Vietnamese students are as follows: TAI 10 – BDI 17 (r = −0.37), TAI 2 – BDI 4 (r = 0.35), TAI 20 – BDI 12 (r = −0.34), TAI 13 – BDI 7 (r = 0.31), and TAI 10 – BDI 19 (r = −0.3). On the other hand, the strongest edges for Chinese students in the moderate test anxiety–depression group are TAI 12 – BDI 20 (r = 0.2), TAI 6 – BDI 21 (r = −0.18), TAI 11 – BDI 8 (r = 0.18), TAI 19 – BDI 20 (r = 0.18), and TAI 20 – BDI 1 (r = 0.18). In the high test anxiety-depression group, the strongest edges for Vietnamese students are TAI 3 – BDI 16 (r = 0.25), TAI 9 – BDI 14 (r = 0.25), and TAI 8–BDI 8 (r = 0.24). Moreover, the strongest edges for Chinese students in the same group are TAI 7 – BDI 3 (r = 0.35), TAI 7 – BDI 16 (r = 0.33), TAI 9 – BDI 17 (r = 0.33), and TAI 3 – BDI 16 (r = 0.32) (see Figure 2).

## 4 Discussion

This study utilized the Test Anxiety Inventory (TAI) and the Beck Depression Inventory-II (BDI-II) to conduct a comparative investigation of the comorbidity between test anxiety and depression among Chinese and Vietnamese university students, employing multi-group latent profile analysis and network analysis. The results from the multi-group latent profile analysis indicate that the TAI and BDI-II demonstrate equivalence across the cultural contexts of China and Vietnam, exhibiting similar latent structural characteristics (Fan et al., 2019). The network analysis findings reveal that both Chinese and Vietnamese undergraduate students experience comorbid test anxiety and depression, although the core manifestations differ slightly.

Our results indicate that among Vietnamese students, test anxiety and depression mainly manifest as negative emotional states, including low mood, physical exhaustion, helplessness, worthlessness, and fatigue. Heightened tension can lead to forgetfulness, hindering recall during exams. In contrast, Chinese students exhibit a pattern characterized by heightened tension and anxiety, intense emotional strain, panic, restlessness, and persistent worry about failure. This psychological pressure affects their emotional state and results in cognitive impairments, such as zoning out, freezing, or becoming easily distracted, which aligns with findings from Hou et al. (2025) and Zhang et al. (2019).

Significant differences exist in the connections between symptoms of comorbid test anxiety and depression in China and Vietnam. Vietnamese students tend to exhibit higher levels of depressive tendencies, whereas Chinese students show a significant positive association between test anxiety and depression. Additionally, the test anxiety–depression network among Chinese students is more tightly connected (see Figure 2), indicating a greater risk of comorbidity. Furthermore, symptom connections at different levels of test anxiety and depression vary between the two countries.

The main differences in test anxiety and depression comorbidity between China and Vietnam are clearly evident in the moderate test anxiety–depression and high test anxiety–depression groups. In the moderate test anxiety–depression network, although the associations between test anxiety and depressive symptoms are slightly stronger among Vietnamese students than among their Chinese counterparts, the connections remain relatively weak overall. Among Chinese students, the more they feel troubled during an exam, the more they experience fatigue. They are also likely to feel guilty due to their anxiety, with this guilt becoming more pronounced when they reflect on the exam afterwards, leading to greater exhaustion. Additionally, when tension causes them to forget previously learned material, they tend to feel especially sad. Even if Vietnamese students feel anxious before submitting their papers, they are unlikely to become angry. Similarly, forgetting material due to tension does not diminish interest. Despite feeling anxious as exams approach, their focus tends to remain intact. However, they may feel disappointed by physical discomfort during exams and lose enjoyment because of exam-related stress. In the high test anxiety–depression network, both Chinese and Vietnamese students exhibited positive correlations between test anxiety and depressive symptoms. At this stage, students in both countries experience sleep disturbances due to worries about their grades. Moreover, Chinese students also reported sleep problems related to concerns about poor exam performance, a pattern not observed among Vietnamese students. When students feel extremely anxious despite being well prepared, their responses differ: Chinese students may become angry, whereas Vietnamese students are more likely to experience feelings of worthlessness. If Vietnamese students feel unsettled during an exam, they tend to engage in self-blame, whereas Chinese students are more prone to feelings of failure because of their inability to concentrate. While the test anxiety–depression networks of both countries exhibit some similarities at high anxiety–depression levels, distinct characteristics emerge at low–moderate levels. Notably, Vietnamese students display better emotional resilience in low-to-moderate anxiety-depression networks. This difference may be linked to emotional regulation strategies and how individuals interpret mental illness (Kleinman and Eisenberg, 1987). They also note that, unlike disease, which is viewed as a biological malfunction, psychological distress—such as test anxiety and depressive tendencies in this case—can be seen as a precursor to mental illness, influenced by cultural values and social norms that shape how individuals perceive, label, explain, and evaluate their discomforting experiences. This interplay of personal and cultural factors suggests that mental health is not only a biological phenomenon but also a social construct, whose expression and interpretation can vary significantly across cultures. In line with this, Kramer et al. (2002) found that cultural understandings of mental health differ significantly across countries. For example, although mental health issues are stigmatized to varying degrees in both Vietnamese and Chinese cultures, Vietnamese culture tends to view mental illness more as an emotional struggle (e.g., “Depression is sadness”), whereas Chinese culture is more likely to interpret it as an emotional imbalance or even supernatural interference (e.g., by evil spirits) (Kramer et al., 2002). These perceptions influence how individuals and families respond to psychological distress. In Vietnam, people tend to seek help from those around them before turning to professional intervention, with family and friends often providing support through distraction or humor. In contrast, Chinese people may prioritize traditional treatments, such as herbal medicine or exorcism rituals, although the effectiveness of these methods in alleviating psychological disorders remains uncertain.

Another factor contributing to these differences may be the overall pressure exerted by the educational systems in the two countries. China is well known for its highly competitive exam culture, which places greater social expectations on male students than Vietnam does (Blum et al., 2012; Thanh et al., 2006). Research by Carlessa and Lam (2014) found significant academic competition among lower primary school students, with teachers seemingly encouraging such comparisons. This is consistent with the high masculinity of Chinese society, where competition, achievement, and success are key societal drivers (Lee and Ande, 2023). Influenced by Confucian cultural values, Chinese culture prioritizes males and the eldest in hierarchical structures, placing high expectations on their academic achievements (Jin et al., 2024; Stankov, 2010). This aligns with findings from Nyroos et al. (2015) and Sharma and Sud (1990). In contrast, Vietnam has one of the most feminine cultures in the world, with a Hofstede femininity score of 40 (Lee and Ande, 2023). This suggests that societal values prioritize concern for others, emotional support, and quality of life over individual competitiveness, which may help protect individuals from severe mental health issues and prevent the exacerbation of psychological distress caused by academic competition.

Both China and Vietnam are influenced by collectivist and Confucian cultures, which impose varying degrees of academic pressure (Vượng et al., 2015). However, while Confucianism is deeply rooted in Chinese culture, Vietnamese society has integrated Buddhist principles more effectively (Hanh, 2001; Vượng et al., 2015). Some researchers suggest that the influence of Buddhism in Vietnamese culture may act as a natural buffer against psychological disorders (Blum et al., 2012; Thanh et al., 2006). Moreover, despite the lower social status of women in Vietnamese society, their significant contributions to social and economic life—particularly through their roles in the traditional “small business” sector—and the profound influence of matrilineal traditions have shaped a paradoxical status for modern Vietnamese women (Vượng et al., 2015). This dynamic, often encapsulated in the saying “the husband rules, the wife governs” (le mari règne, la femme gouverne), may further exacerbate the academic pressures faced by Vietnamese women (Thảo and Ái, 2021).

The results of the latent profile analysis indicate that the levels of test anxiety differ between China and Vietnam across different stages. Specifically, in the low test anxiety-depression group, Vietnamese students exhibited higher levels of test anxiety than their Chinese counterparts. However, in the high test anxiety-depression group, the test anxiety levels of Vietnamese students were lower than those of Chinese students. This pattern suggests that although Chinese students experience more severe test anxiety, the prevalence of test anxiety symptoms is greater among Vietnamese students (with detection rates of 13.57% and 31.9%, respectively). These differences may be related to the social acceptability of behaviors (Blum et al., 2012), emotional sensitivity (Fei et al., 2018) and Vietnam’s higher indulgence score, which reflects a greater societal tolerance for emotional expression compared to China (Kramer et al., 2002; Lee and Ande, 2023). However, to better understand this phenomenon and the factors contributing to it, further research is needed.

In this study, we employed multi-group latent profile analysis and network analysis for the first time to explore the comorbidity of test anxiety and depression within the cultural contexts of China and Vietnam. The participants were primarily undergraduate students, meaning that the findings may not be representative of the entire student population or students from different regions. Furthermore, the study was designed as a cross-sectional investigation, which may limit our understanding of temporal changes (especially during the pandemic) and causal relationships. Although this study provides valuable insights, it does not fully account for the complexity and diversity of Chinese and Vietnamese sociocultural factors and their potential impact on individuals’ mental health. Future research could explore these elements further to develop a more comprehensive understanding of how test anxiety and depression manifest in different cultural contexts and the factors influencing their development.

## 5 Conclusion

Both Chinese and Vietnamese university students experience the comorbidity of test anxiety and depression. However, Chinese students with high test anxiety tend to exhibit stronger depressive tendencies. The core symptoms of comorbid test anxiety and depression differ between the two groups. Chinese students frequently experience more intense emotional tension and anxiety, whereas Vietnamese students are more prone to negative emotional states. While the test anxiety–depression networks in both countries show some similarities at high anxiety–depression levels, distinct characteristics emerge at low–moderate levels of test anxiety and depression.

## Data Availability

The raw data supporting the conclusions of this article will be made available by the authors, without undue reservation.
